# Black-box and surrogate optimization for tuning spiking neural models of striatum plasticity

**DOI:** 10.3389/fninf.2022.1017222

**Published:** 2022-10-20

**Authors:** Nicolás C. Cruz, Álvaro González-Redondo, Juana L. Redondo, Jesús A. Garrido, Eva M. Ortigosa, Pilar M. Ortigosa

**Affiliations:** ^1^Department of Computer Engineering, Automation and Robotics, University of Granada, Granada, Spain; ^2^Department of Informatics, University of Almería, ceiA3 Excellence Agri-food Campus, Almeria, Spain

**Keywords:** model tuning, surrogate optimization, black-box optimization, striatum, reinforcement learning, spiking neural networks, dopamine, spike-timing dependent plasticity (STDP)

## Abstract

The basal ganglia (BG) is a brain structure that has long been proposed to play an essential role in action selection, and theoretical models of spiking neurons have tried to explain how the BG solves this problem. A recently proposed functional and biologically inspired network model of the striatum (an important nucleus of the BG) is based on spike-timing-dependent eligibility (STDE) and captured important experimental features of this nucleus. The model can recognize complex input patterns and consistently choose rewarded actions to respond to such sensory inputs. However, model tuning is challenging due to two main reasons. The first is the expert knowledge required, resulting in tedious and potentially biased trial-and-error procedures. The second is the computational cost of assessing model configurations (approximately 1.78 h per evaluation). This study addresses the model tuning problem through numerical optimization. Considering the cost of assessing solutions, the selected methods stand out due to their low requirements for solution evaluations and compatibility with high-performance computing. They are the SurrogateOpt solver of Matlab and the RBFOpt library, both based on radial basis function approximations, and DIRECT-GL, an enhanced version of the widespread black-box optimizer DIRECT. Besides, a parallel random search serves as a baseline reference of the outcome of opting for sophisticated methods. SurrogateOpt turns out to be the best option for tuning this kind of model. It outperforms, on average, the quality of the configuration found by an expert and works significantly faster and autonomously. RBFOpt and the random search share the second position, but their average results are below the option found by hand. Finally, DIRECT-GL follows this line becoming the worst-performing method.

## 1. Introduction

Computational models of the brain are useful tools for learning mechanisms. However, the difficulty involved in finding parameters that provide good solutions is a major challenge.

A model already published in a previous article (Gonzalez-Redondo et al., 2022) is a complex model that is difficult to obtain good solutions for. This model tries to better understand how learning through interaction to achieve a goal is solved by animals (or agents) by choosing among many possible actions to obtain rewards, as described in the reinforcement learning (RL) paradigm (Sutton et al., 1992). The model is based on spike-timing-dependent eligibility (Gurney et al., 2015) (STDE), a learning rule capturing important experimental features in the brain and, specifically the basal ganglia (BG, a set of nuclei located in the forebrain). This brain structure is related to the process of action-selection, according to biological (Graybiel, 1998; Hikosaka et al., 2000; Grillner et al., 2005) and computational studies (Redgrave et al., 1999; Gurney et al., 2001; Tomkins et al., 2014). We implemented (Gonzalez-Redondo et al., 2022) a functional and biologically inspired network model of the striatum (STR, an important input nucleus of the BG), where learning is based on STDE. The proposed model has been demonstrated to be capable of recognizing input patterns relevant to the task and consistently choosing rewarded actions in response to that input.

However, models require tuning (Van Geit et al., 2007; Mart́ınez-Álvarez et al., 2016), and the quality expectations, datasets, and adaptability requirements are continuously growing (Van Geit et al., 2008; Masoli et al., 2020). The model described in Gonzalez-Redondo et al.'s (2022) study, which attracts the attention of this work, contains dozens of free parameters: learning kernel shapes, synaptic and neuron time constants, lateral inhibition weight, etc. Some of them can be inferred from experimental data, but most of them must be manually tuned with plausible values. With this number of parameters, the curse of dimensionality leads to a tedious trial-and-error search procedure prone to failures. Another problem is the computational cost of evaluating each model configuration: it takes approximately 1.78 h per evaluation in a modern laptop using a single CPU core. Both made the tuning of our model slow (the parameters finally used were found after 2 months of search), sub-optimal (as there is a huge parametric space not covered), and biased (by the intuition of the expert). Fortunately, model tuning can be addressed as a global optimization problem. There exists modern frameworks, such as Ray[Tune] (Liaw et al., 2018) and Vizier (Golovin et al., 2017), which implement multiple algorithms compatible with this purpose. Besides, the current increase in computer power that allows for defining more sophisticated models also helps us to face more challenging optimization problems (Van Geit et al., 2008; Cruz et al., 2021; Maŕın et al., 2021).

When addressing model tuning as an optimization problem, the objective function generally represents the difference between the desired and achieved output of the model for any candidate configuration. Concerning the associated problem, when the objective function exhibits mathematically exploitable properties, such as linearity, convexity, and continuous variables, it can be exactly solved. Otherwise, its resolution can be significantly challenging (Lindfield and Penny, 2017; Salhi, 2017). This issue might arise when the objective function does not have a closed analytical form or relies on sophisticated models with non-linear expressions, uncertainty, and simulations (Cruz et al., 2018; Maŕın et al., 2021). Luckily, some methods aim at finding acceptable results with a reasonable effort by using randomness and intuitive ideas. Most heuristics and meta-heuristics would fall into this group (Lindfield and Penny, 2017; Salhi, 2017). Similarly, if a method does not have specific knowledge or strict requirements for the objective function apart from being able to evaluate candidate solutions, it is classified as a black-box optimizer (Audet and Hare, 2017; Golovin et al., 2017). Both categories are frequently linked, as many meta-heuristics, such as evolutionary and swarm intelligence algorithms, are also black-box methods.

In this context, black-box optimization methods can be classified into two groups: those without specific components to require few function evaluations and those with them. It could be said that needing a few function evaluations to converge is one of the goals pursued when designing any optimization method. However, most population-based meta-heuristics need numerous function evaluations (Costa and Nannicini, 2018) to compensate for their instability due to randomness (Jones and Martins, 2021). They would hence fall into the first group. For instance, for the successful evolutionary optimizer UEGO (Garćıa-Mart́ınez et al., 2015; Cruz et al., 2018; Maŕın et al., 2021), a robust configuration could need up to 1,000,000 function evaluations (Ortigosa et al., 2001). This potential requirement is usually attenuated with parallel computing, which fits well with population-based algorithms (Storn and Price, 1997; Jelásity, 2013; Cruz et al., 2019). This can be seen as a brute-force approach to tackle the high consumption of function evaluations. The methods in the second group do not renounce the benefits of high-performance computing, but they try to avoid function evaluations by design. Their use can be the only option when the cost of evaluating the objective function cannot be hidden with parallel computing. The most relevant methods in this group are surrogate optimizers (Vu et al., 2017; Bhosekar and Ierapetritou, 2018; Costa and Nannicini, 2018), which avoid evaluating the real objective function by constructing a lightweight model of it. They define an active research line in global optimization.

In this work, the objective function is not a plain mathematical function, such as a parabola. Instead, each evaluation launches a process that consists of building the neural network according to the input parameters of the candidate configuration, training it, and returning its performance at the target task. As mentioned above, this process is computationally demanding. For this reason, this study pays attention to optimization algorithms requiring few function evaluations. The selection consists of four solvers in total. The first two are SurrogateOpt, provided by the official Global Optimization Toolbox of Matlab (López, 2014), and RBFOpt (Costa and Nannicini, 2018), open-sourced and written in Python. Both constructed a surrogate of the real objective function by combining radial basis ones (Gutmann, 2001; Regis and Shoemaker, 2007). The third method is DIRECT-GL (Stripinis et al., 2018), an enhanced version of the widespread DIRECT (Jones and Martins, 2021), which stands out due to its deterministic and effective strategy of dividing the search space and prioritizing the most promising areas to save function evaluations. The last one is a simple random search (Cruz et al., 2018), which is expected to define the baseline performance. Nonetheless, this method has also been implemented to benefit from parallel computing, so its rate of the evaluation of candidate solution is high. To the best of the authors' knowledge, the tuning of spiking neural models of striatum plasticity has not been studied from this perspective before. Hence, the ultimate goal of this research is to recommend the most effective strategy to save tedious trial-and-error procedures and hyper-parameter tuning for spiking neural networks (SNNs) by hand in general, which is inherently biased by the expert.

The rest of the paper is structured as follows: Section 2 describes materials and methods. Section 3 contains the experimentation and results. Finally, Section 4 shows the conclusions and states future work.

## 2. Materials and methods

This section starts with a detailed description of the computational models that define the agent behavior and the task it is solving. After that, the four optimization strategies considered are explained.

### 2.1. Computational models

For the network model, we used conductance-based versions of the leaky-integrate and fire (LIF) neuron model (Gerstner and Kistler, 2002). LIF model simplifies many aspects of neuronal dynamics, thus it is more computationally efficient than other commonly used neural models in SNNs. We used this model in every layer of the network. Before the use of optimization methods, the parameters were manually tuned to obtain reasonable firing rates (see details in Supplementary materials). The STR neurons are divided into D1 or D2 populations, each one with different learning kernel constants (so they can learn to respond to different situations; more on this later) and also divided by channels (one per action). STR D1 and D2 populations are complementary, as the D1 population tries to learn what action it has to do, while D2 population tries to learn what action it has to stop. The action neurons are a population that integrates its channel activity and outputs the behavior of the agent, and they are tuned to fire every input cycle if they receive enough stimulation (at least two more spikes from D1 neurons than D2 neurons each cycle). The dopamine neuron was tuned to have a firing range from 50 to 350 spikes per second, with these unrealistic values chosen to improve computational performance (instead of simulating a bigger dopaminergic population).

**Figure 1:**
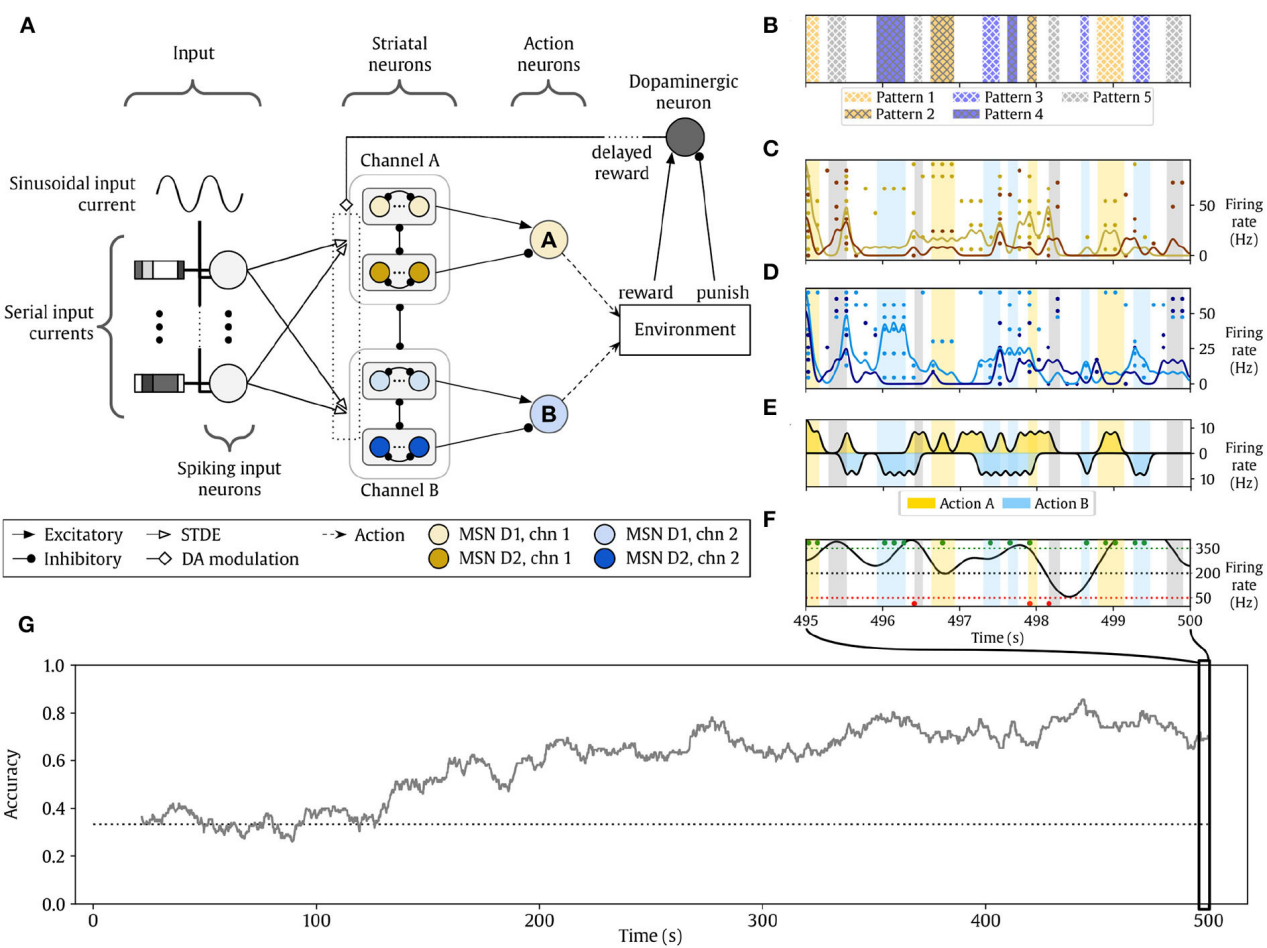
Cortico-striatal network solving a reinforcement learning (RL) task (from Gonzalez-Redondo et al. 2022). **(A)** Structure of the network. **(B–F)** The activity of the network during the last 5 s of simulation. Background color indicates the reward policy (yellowish colors, action A is rewarded and B is punished; bluish colors, action B is rewarded and A is punished; gray, any action is punished). **(B)** Input pattern conveyed to the input layer. **(C)** Raster plot of the channel-A action neurons. Yellow dots represent STR D1 spikes, and orange dots are STR D2 spikes. **(D)** Raster plot of channel B. Cyan dots represent STR D1 spikes, and dark blue dots are STR D2 spikes. **(E)** Action neuron firing rates. The middle horizontal line represents 0 Hz. Action A and B activities are represented in opposite directions for clarity. Action A neuronal activity increases in yellow zones while action B neuronal activity in cyan intervals. **(F)** Firing rate of the dopaminergic neuron (black line). Dotted horizontal lines indicate the range of dopamine activity considered: black is the baseline, green is the maximum reward, and red represents the maximum punishment. Dots indicate rewards (green) and punishment (red) events delivered to the agent. **(G)** Evolution of the learning accuracy of the agent, see Section 2.1 for further details. The dotted line marks the accuracy level by chance.

The input generation procedure is described in Gonzalez-Redondo et al. (2022) and based on Masquelier et al. (2009), Garrido et al. (2016). The agent perceives the environment as 2,000 analog inputs. These inputs are fed one-to-one to an input layer of LIF neurons as currents (Figure 1B), altogether with an oscillatory drive. This oscillatory drive leads to a current-to-phase conversion: the neurons that receive the strongest analog input currents will fire first during the phase of the cycle (Masquelier et al., 2009). This way, we encode analog inputs in specific spatio-temporal spike activity patterns. This is called phase-of-firing encoding and represents information in the spike times of neurons relative to the phase of a background oscillation (in our case, the oscillatory drive). New input stimuli are presented at uniformly distributed random intervals of 200–500 ms. The stimulus can be a repeating pattern or noise, and both are generated randomly depending on the simulation seed. When presenting a repeating pattern, only half of the input neurons (1,000) are pattern-specific, while the other half receives random current values. When no pattern is presented, all the input neurons receive random current values.

The network model (Figure 1A) contains two channels. Every channel contains two parallel layers (STR D1 and D2 neurons, respectively) of striatal-like neurons with asymmetrical structured lateral inhibition (as in Burke et al., 2017) within and between STR D1 and D2 populations. The output of each channel is an action node that integrates the channel activity to decide if the agent takes an action or not. The agent can do none, both, or any of them at a time. A dopaminergic neuron projects its activity to both action channels as a neuromodulator (dopamine) determining what the agent should learn from the recent past experience. An environment reward signal (based on the chosen and the expected action) is delivered to this neuron as excitatory (rewards) or inhibitory (punishments) inputs.

**Figure 2:**
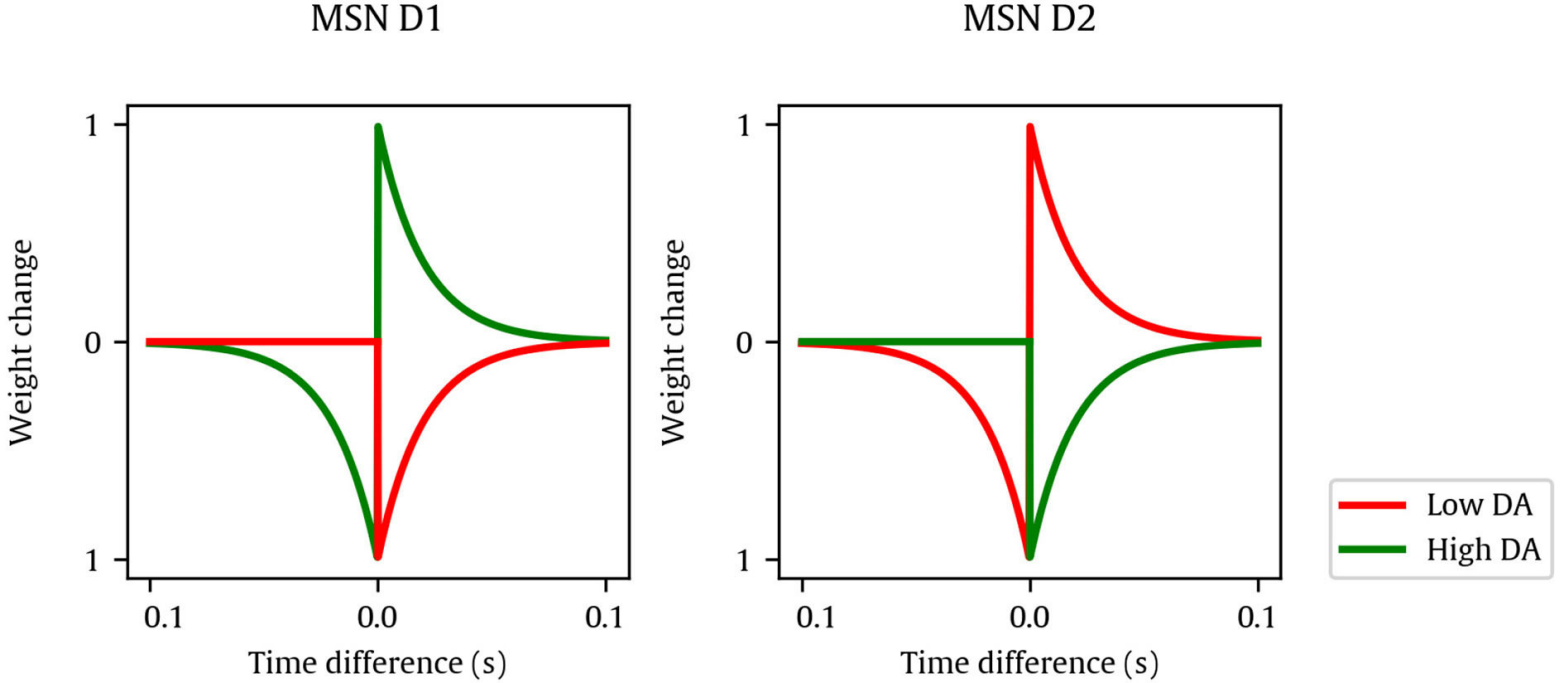
Manually-tuned kernels used for spike-timing-dependent eligibility (STDE) synapses of MSN D1 **(left)** and D2 **(right)**, showing the weight change depending on the time difference between pre- and post-synaptic spikes and dopamine. Lines represent kernels at dopamine minimum and maximum values (red and green, respectively).

The neurons in each channel receive plastic synapses from the input layer. The STDE (Gurney et al., 2015) learning rule is used, a modification of a reward-modulated STDP learning rule where the kernel constants are dopamine-dependent (that is, different values are defined for low dopamine and high dopamine values, see Figure 2). This rule also uses eligibility traces to store the potential changes, similarly to Izhikevich (2007). The learning kernels are different for STR D1 and D2 neurons, as their biological counterparts respond to different situations (Gerfen and Surmeier, 2011): D1 neurons are more predominant in the direct pathway of the BG, which tend to promote behavior when it is active. D2 neurons are more predominant in the indirect pathway of the BG, which tends to inhibit behavior when it is active. For this reason, the initial learning kernels were manually chosen to be complementary: D1 neurons learn to do actions, and D2 neurons learn to stop actions. The dopaminergic modulatory signal is global and delivered to every STDE connection from the input layer to channel neurons. Lastly, two homeostatic mechanisms are added to improve the learning process: first, the synapses implementing the STDE included a non-Hebbian strengthening in response to every pre-synaptic spike. Second, we included adaptive threshold to our neuron models based on Galindo et al. (2020).

The agent has to learn a simple mapping task from stimulus to action. Every 200–500 ms, a new stimulus is presented, and the agent has to respond with the appropriate action. There are five different repeating patterns, and the agent has two possible actions to choose, A or B. Two patterns require action A, the other two patterns require action B, and the fifth pattern requires to do nothing. If the agent responds correctly, the environment gives a reward. If a different action is taken, a punishment is given. If the input is just noise, the environment does not give rewards or punishments.

We used a confusion matrix to help measure the performance of the model. Each row indicates the rewarded action in response to the presented pattern, and each column indicates the selected action in response to the presented pattern. Every cell *m*_*ij*_ then counts the number of occurrences of *j* action being done when *i* action was expected to be done. We only considered in the calculation those trials in which some reward or punishment can be delivered, ignoring those intervals with only noise as the stimulus. We considered that an action has been taken if the corresponding action neuron has spiked at least once during the pattern presentation, and conversely, we consider that no action has been taken if none of the action neurons spikes during the same duration. By doing so, we obtained a confusion matrix, widely used in classification problems when the objective is to describe the accuracy of a final map process (Stehman, 1997). The confusion matrix is defined as in expression (1),


(1) $$\begin{array}{l} C\equiv \left[\begin{array}{cccc} m_{11} & m_{12} & \cdots & m_{1C}\\ m_{21} & m_{22} & \cdots & m_{2C}\\ \vdots & \vdots & \ddots & \vdots \\ m_{C1} & m_{C2} & \cdots & m_{CC} \end{array}\right] \end{array}$$

where *m*_*ij*_ represents the number of occurrences belonging to the *i*-th class (the rewarded action) but classified as members of the *j*-th class (the selected action).

We then measured the model's performance as the accuracy of the classification, which is defined as the sum of the number of correct predictions (the trace of the matrix) divided by the total number of pattern presentations considered (the sum of the whole matrix). In order to measure the evolution through time of the performance of the models, we calculated the confusion matrix for each pattern presentation and then used a rolling mean of the last 100 values to obtain an estimation of the temporal evolution of the accuracy.

### 2.2. Model tuning as an optimization problem

In this context, it is possible to measure the performance of the model resulting from any set of parameters as the accuracy, *F*, of the classification, according to Equation (2). This value is defined as the sum of the number of correct predictions (the trace of the matrix) divided by the total number of pattern presentations considered (the sum of the whole matrix). To measure the evolution through time of the performance of the models, we calculated the confusion matrix for each pattern presentation and then used a rolling mean of the last 100 values to obtain an estimation of the temporal evolution of the accuracy.


(2) $$\begin{array}{l} F=\frac{\sum_{i}m_{ii}}{\sum_{i}\sum_{j}m_{ij}} \end{array}$$

The value of *F* was ultimately dependent on 13 variables, a selected subset of all the variables of the model, which determined the model behavior. Notice that the model features inherent stochasticity, which is handled by returning the average of five simulations. The variables are shown in Table 1, including their corresponding ranges. These variables have been chosen to be optimized as they are the ones related to the learning process of the model. We did not optimize the neuron model variables as we already found reasonable values to make their firing behavior match their biological counterparts. Variable *w*_*max*_ represents the maximum weight of each plastic synapse. Variable *C*_*pre*_ is the homeostatic term applied per presynaptic spike. Variable τ_*th*_ is the time constant of the adaptive neuron threshold. Variable *C*_*th*_ defines the additive increment of the adaptive threshold of a striatal neuron after a spike, and it is inversely proportional to the target firing rate. Variable μ is a dimensionless constant that modulates all learning parameters. Lastly, the kernel shape of the STDE learning rule is defined by the parameters $k_{DA}^{SPK}$ with *SPK*∈{+, −} being the spike order pre-post for applying $k_{DA}^{+}$ and post-pre for applying $k_{DA}^{-}$, respectively, and *DA*∈{*hi, lo*} being the high- or low-DA cases, resulting in four parameters per neuron population: $k_{hi}^{+}$, $k_{lo}^{+}$, $k_{hi}^{-}$ and $k_{lo}^{-}$. As we have two neuron populations *POP*∈{*d*1, *d*2}, there are eight ${k_{POP}}_{DA}^{SPK}$ STDE parameters in total: ${k_{d1}}_{hi}^{+}$, ${k_{d1}}_{lo}^{+}$, ${k_{d1}}_{hi}^{-}$, ${k_{d1}}_{lo}^{-}$, ${k_{d2}}_{hi}^{+}$, ${k_{d2}}_{lo}^{+}$, ${k_{d2}}_{hi}^{-}$, and ${k_{d2}}_{lo}^{-}$. A graphical representation of all these kernels for the manually-tuned case can be found in Figure 2.

**Table 1:** Parameters to tune for the neural model and their allowed ranges.

**Variable**	**Lower bound**	**Upper bound**	**Unit**
*w*_*max*_	10^−3^	10^−1^	μS
*C*_*pre*_	−10^−5^	10^−5^	μS
μ	5·10^−4^	5·10^−2^	-
τ_*th*_	1	200	s
*C*_*th*_	10^−2^	2	mV
${k_{d1}}_{hi}^{+}$	−1	1	-
${k_{d1}}_{hi}^{-}$	−1	1	-
${k_{d1}}_{lo}^{+}$	−1	1	-
${k_{d1}}_{lo}^{-}$	−1	1	-
${k_{d2}}_{hi}^{+}$	−1	1	-
${k_{d2}}_{hi}^{-}$	−1	1	-
${k_{d2}}_{lo}^{+}$	−1	1	-
${k_{d2}}_{lo}^{-}$	−1	1	-

Based on this quality metric and the variables involved, model tuning can be expressed as an optimization problem. It focuses on finding the values of the parameters (within their feasible range) that maximize the value of *F*, which becomes the objective function in optimization terms. The problem can be formulated according to Equation (3). For simplicity, only the first and the last variables are shown. The constraints keep every variable in its feasible range, which results in a box-constrained problem (Costa and Nannicini, 2018; Stripinis and Paulavičius, 2022). The max and min superscripts linked to each parameter symbol denoted its upper and lower bounds, respectively. The numerical values are those shown in Table 1.


(3) $$\begin{array}{l} \underset{w_{max},\ldots ,{k_{d2}}_{lo}^{-}}{\text{maximize}}\;F(w_{max},\ldots ,{k_{d2}}_{lo}^{-})\\ \text{subject to }w_{max}^{lower}\leq w_{max}\leq w_{max}^{upper}\\ \;\ldots \;\\ \;{k_{d2}}_{lo}^{-,lower}\leq {k_{d2}}_{lo}^{-}\leq {k_{d2}}_{lo}^{-,upper} \end{array}$$

Notice that the problem is defined as a maximization one, but optimization methods traditionally aim at minimization. Regardless, this is not relevant, because converting a maximization problem into a minimization one is trivial. It is only necessary to multiply the objective function by –1, i.e., maximizing *F*(… ) is equal to minimizing −*F*(… ).

### 2.3. Optimization methods

As introduced, evaluating the objective function relies on non-deterministic simulations and is computationally demanding. Thus, the methods considered are designed for black-box optimization (Audet and Hare, 2017), i.e., RBFOpt (Costa and Nannicini, 2018), SurrogateOpt (Matlab, 2021), DIRECT-GL (Stripinis et al., 2018), and a random search (Cruz et al., 2018). The first three, which are also the preferred options, have been explicitly designed to require some function evaluations. All of them are prepared for exploiting parallel computing. Finally, it is relevant to highlight that among the considered methods, DIRECT-GL is the only deterministic one, which means that the algorithm does not rely on randomness and always returns the same result for the same problem instance and configuration.

#### 2.3.1. RBFOpt

RBFOpt, published in Costa and Nannicini (2018), is an open-source library written in Python for black-box optimization with computationally-expensive objective functions. This tool is based on the method proposed by Gutmann (2001).

RBFOpt belongs to the family of surrogate optimization methods. The fundamental idea of surrogate optimization is that the process relies on iteratively building an approximate model (response surface or surrogate model) of the real objective function. While the former approximates the latter, its computational requirements are expected to be significantly lower, and the accuracy can improve as the information on the target function increases with the points evaluated (Vu et al., 2017). For building the surrogate model, RBFOpt uses radial basis functions, whose output depends on the distance between the input and a given reference. In this field, Gutmann (2001) was a pioneer in using radial basis functions for optimizing computationally demanding black-box functions (Costa and Nannicini, 2018).

Let *f*(*x*) be an abstract objective function of form *f* : ℝ^*N*^ → ℝ, where *x* ∈ [*x*^*min*^, *x*^*max*^], and *x*^*min*^, *x*^*max*^ ∈ ℝ^*N*^, i.e., the corresponding lower and upper bounds of each decision variable. Notice that this can be seen as a generalization of the particular problem formulation in Equation (3). For *K* different points of the search space, *x*_1_, …, *x*_*K*_, with known values, *y*_1_ = *f*(*x*_1_), …, *y*_*K*_ = *f*(*x*_*K*_), the associated radial basis function interpolant, *s*_*K*_, has the following structure as the sum of *K* radial basis functions (Vu et al., 2017; Costa and Nannicini, 2018):


(4) $$\begin{array}{l} s_{K}\left(x\right)=\overset{K}{\underset{i=1}{\sum }}\lambda_{i}\phi \left(\left||x-x_{i}|\right|\right)+p\left(x\right) \end{array}$$

where ϕ : ℝ_+_ → ℝ, which is a radial basis function, λ_1_, …, λ_*K*_ ∈ ℝ acting like weights of the model, and *p*(*x*) is a polynomial. The minimum degree of *p* to guarantee the existence of the interpolant depends on the form of ϕ. Table 2 contains three common radial basis functions for a generic input, *r*. It also includes the minimum degree of their accompanying polynomial *p* that ensures the existence of the interpolant. If these components are appropriately configured, the desired radial basis function interpolant can be efficiently computed by solving a linear system to find the unknown parameters, such as the weights. For instance, *x*_1_, …, *x*_*K*_ should be pairwise distinct (Vu et al., 2017; Costa and Nannicini, 2018).

**Table 2:** Frequent radial basis functions.

**ϕ(*r*)**	**Type**	**Minimum degree**
*r*	Linear	0
*r* ^3^	Cubic	1
*r*^2^log*r*	Thin plate spline	1

**Figure 3:**
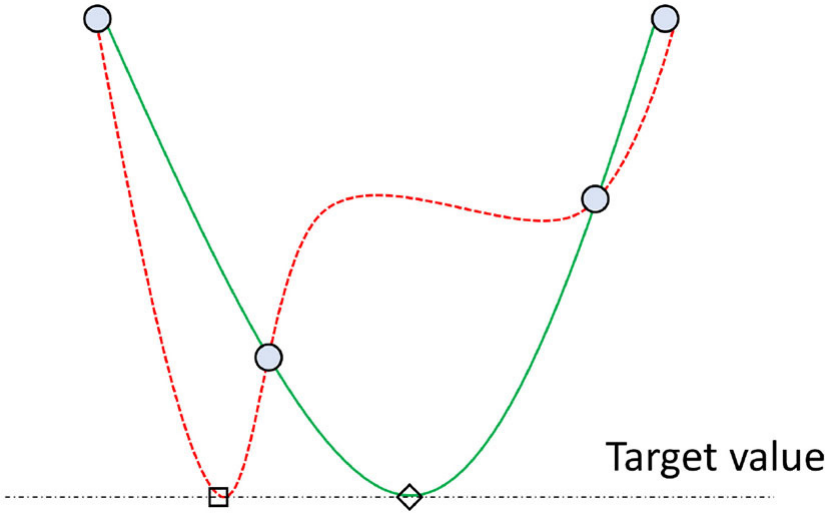
Depiction of two surrogate models interpolating four points (blue circles) and reaching a target value (horizontal dashed line). The green solid-line model is considered more likely than the red dashed-line one since it is smoother. In other words, the method by Gutmann (2001) assumes that it is more likely that the point tagged with a diamond exists (green line) rather than that with a square (red line) in the real function (Costa and Nannicini, 2018).

The general procedure applied by optimization methods using radial basis functions follows Algorithm 1 (Costa and Nannicini, 2018). A particular method will define a strategy to implement these generic steps, starting from selecting the initial points. For example, for low-dimensional problems, a valid approach is to choose the corners of the search space. Another one is to pick the corners and the central point. Controlling the effort put into improving the accuracy of the surrogate model and finding the best point with the current model is also critical. The method by Gutmann (2001) defined a measure of the bumpiness of the surrogate model for this purpose. Their method assumes that the real objective function does not oscillate excessively, thus when configuring models and considering new points, the smoother (or “least bumpy”) interpolant is preferred (see Figure 3, which assumes four known points and a hypothetical target value of the cost function). Regardless, describing these aspects in detail is out of the scope of this paper. Refer to the work by Vu et al. (2017) to have a detailed overview and that by Costa and Nannicini (2018) to understand the fundamentals of RBFOpt.

**Algorithm 1:** Generic global optimization through radial basis functions

1: Initial step - Select *K* points
2: **while** There is available function evaluations **do**
3: Compute the radial basis function interpolant
4: Decide between improving the surrogate model and finding the best point using the current model.
5: Determine the next point to consider according to the previous decision
6: Evaluate the objective function at the new point
7: **end** **while**
8: **return** Best point found

In this context, RBFOpt has two main contributions. The first is an automatic model selection component. The second is the support for using faster yet less accurate variants of the objective function. The latter is especially appropriate for the target problem since the simulation-related parts of the objective function, such as the training time and the seeds, are adjustable. They can be modified by the expert in charge of model tuning to reduce time at the expense of losing accuracy. These properties, along with its open-source nature, the compatibility with parallel computing, and the good results reported in Costa and Nannicini (2018) motivated its consideration for the present work.

#### 2.3.2. SurrogateOpt

SurrogateOpt is a solver for computationally-demanding black-box optimization problems provided by the Global Optimization Toolbox (López, 2014) of Matlab (2021) since its version R2018b. As introduced, it belongs to the same group as RBFOpt since the method is a surrogate optimization algorithm. SurrogateOpt also uses radial basis function interpolators. Its documentation motivates this decision by highlighting that they support any number of dimensions and are computationally cheap to construct, evaluate, and extend. This tool is mainly based on the algorithm proposed by Regis and Shoemaker (2007). It has been selected due to its effectiveness, simplicity of use, and compatibility with parallel computing.

Conceptually, SurrogateOpt follows a scheme similar to Algorithm 1. The fundamental differences correspond to the implementation of each step and specific definitions. In this regard, SurrogateOpt has a rich set of associated concepts and procedures, which are summarized below. According to its official documentation, the method alternates between two stages: constructing surrogate and searching for minimum. The change between them occurs after what is called the surrogate reset.

In the first stage, the method builds a surrogate of the real objective function. For this purpose, it interpolates a radial basis function through a set of points whose value must be computed with the real yet computationally demanding objective function. SurrogateOpt uses a cubic radial basis function with a linear tail, which minimizes the concept of bumpiness (Gutmann, 2001), previously mentioned when describing RBFOpt. In the beginning, the solver computes and evaluates a user-given number of random points distributed adequately within the bounds. It can also start from a user-given set of points of known value. In later executions of this stage, the software package will create and evaluate a parameter-defined number of random points. As explained for RBFOpt, building the desired interpolant involves solving a linear system of equations.

In the second stage, SurrogateOpt looks for a minimum of the objective function using a procedure that resembles a local search. More specifically, the method defines a search region radius, known as the scale, whose initial value is 0.2. It starts from the best point since the last surrogate reset, i.e., the one with the smallest objective function value. This point is called the incumbent point. The search then focuses on finding a minimum of merit function that relates the surrogate and the distance from the points evaluated with the real objective function. This approach aims to find a trade-off between minimizing the surrogate, which is not the real objective function and is potentially less accurate, and evaluating new points accurately.

Mathematically, the definition of the merit function for any point *x* combines two weighted terms, the scaled surrogate, *S*(*x*), and the scaled distance, *D*(*x*). Being *s*_*min*_ and *s*_*max*_, the minimum and maximum surrogate values of the sample points, respectively, and *s*(*x*) that of the considered point, the scaled surrogate is defined as follows Matlab (2021):


(5) $$\begin{array}{l} S\left(x\right)=\frac{s\left(x\right)-s_{min}}{s_{max}-s_{min}} \end{array}$$

*S*(*x*) is non-negative and zero at points having minimal surrogate values among sample points. Concerning the scaled distance, it is defined as follows:


(6) $$\begin{array}{l} D\left(x\right)=\frac{d_{max}-d\left(x\right)}{d_{max}-d_{min}} \end{array}$$

where *d*_*min*_ and *d*_*max*_ are the minimum and maximum distances from a sample point to any evaluated one, respectively, and *d*(*x*) is the minimum distance of the point *x* to an evaluated one. *D*(*x*) is non-negative, and zero at points at the furthest distance from evaluated points. Hence, minimizing *D*(*x*) orientates the algorithm toward regions separated from evaluated points. The merit function is a convex combination of both parts according to the following structure:


(7) $$\begin{array}{l} wS\left(x\right)+\left(1-w\right)D\left(x\right) \end{array}$$

where *w* is a weighting factor between zero and one. The greater it is, the most effort is put into minimizing the surrogate model. Analogously, the smaller it is, the most interest is in exploring new regions. This weighting factor cycles through the following values, according to Regis and Shoemaker (2007): 0.3, 0.5, 0.8, and 0.95.

During the search, the solver adds multiple (up to thousands) random vectors to the incumbent point to generate sample points. The vectors are shifted and scaled by the bounds in each dimension and ultimately multiplied by the scale. The sample points must also respect the problem bounds. Then, the merit function is evaluated at all of them further than a parameter-defined distance from any point previously evaluated. The one featuring the best (lowest) value of the merit function becomes an adaptive point. The real objective function will be ultimately computed at it, which will be used to update the surrogate model and assess the real gain from the incumbent value. If the real value of the adaptive point is significantly better than the current incumbent point, the former replaces the latter, and the search is considered successful. Otherwise, the incumbent point remains unaltered, and the search is classified as unsuccessful.

The scale of the search changes when one of the following conditions are met:

1. There have been three successful searches since the last scale change.
2. There have been either five or the number of problem variables (whichever is greater) unsuccessful searches since the last scale change.

If the first condition is met, the scale is doubled (up to a maximum length of 0.8 times the size of the box defined by the problem bounds). If the second situation occurs first, the scale is divided by two (without becoming lower than 1e−5 times the size of the box defined by the problem bounds). By proceeding this way, the search ultimately focuses near an incumbent point featuring a small objective function value.

After considering all the new sample points further than a minimum distance from the evaluated points, the search for minimum phase ends to go back to the construct surrogate one, i.e., resetting the surrogate model. This phase change generally occurs after reducing the scale until all sample points are closely around the incumbent point.

#### 2.3.3. DIRECT-GL

DIRECT-GL, proposed by Stripinis et al. (2018) and Stripinis and Paulavičius (2022), is an enhanced version of a popular method, DIRECT (Jones and Martins, 2021). This new variant is designed as a modification of a specific part of the original method. Hence, it is convenient to start by describing the initial DIRECT and its framework, inherited by the new one.

DIRECT was proposed by Jones et al. (1993) as a modification of Lipschitzian Optimization that did not require specifying a Lipschitz constant, i.e., a bound on the rate of change of the objective function, which cannot be easily computed in real problems (or it may not exist). Aside from keeping a deterministic behavior, the method was simpler, converged faster, and featured a certain degree of compatibility with parallel computing. It was later revised by Jones (2001) to handle not only box or domain constraints and continuous variables but also nonlinear inequality constraints and integer variables. From the beginning, this method was conceived for black-box optimization and situations in which the objective function was time-consuming.

Global optimization algorithms must find a trade-off between exploration and exploitation of the search space (Van Geit et al., 2008). The first term refers to finding unexplored regions, and the second represents the capacity to find the best solution in a known zone (global and local search capabilities, respectively). In Lipschitz optimization, the Lipschitz constant is treated as a weighting factor determining how much emphasis to put into global over local search by indicating where to split the search space into sub-regions. This value must equal or exceed the maximum rate of change of the objective function, so conservative configurations excessively focus on global search. It also makes these methods slow to converge because modifying the value at search is challenging. In contrast to them, DIRECT could maintain the scheme of dividing the search space and autonomously prioritizing the regions to explore by virtually considering all possible constants. The division was also independent of the number of dimensions, so the algorithm was more scalable with the problem dimensionality (yet not recommended for more than 20 variables Jones, 2001).

**Figure 4:**
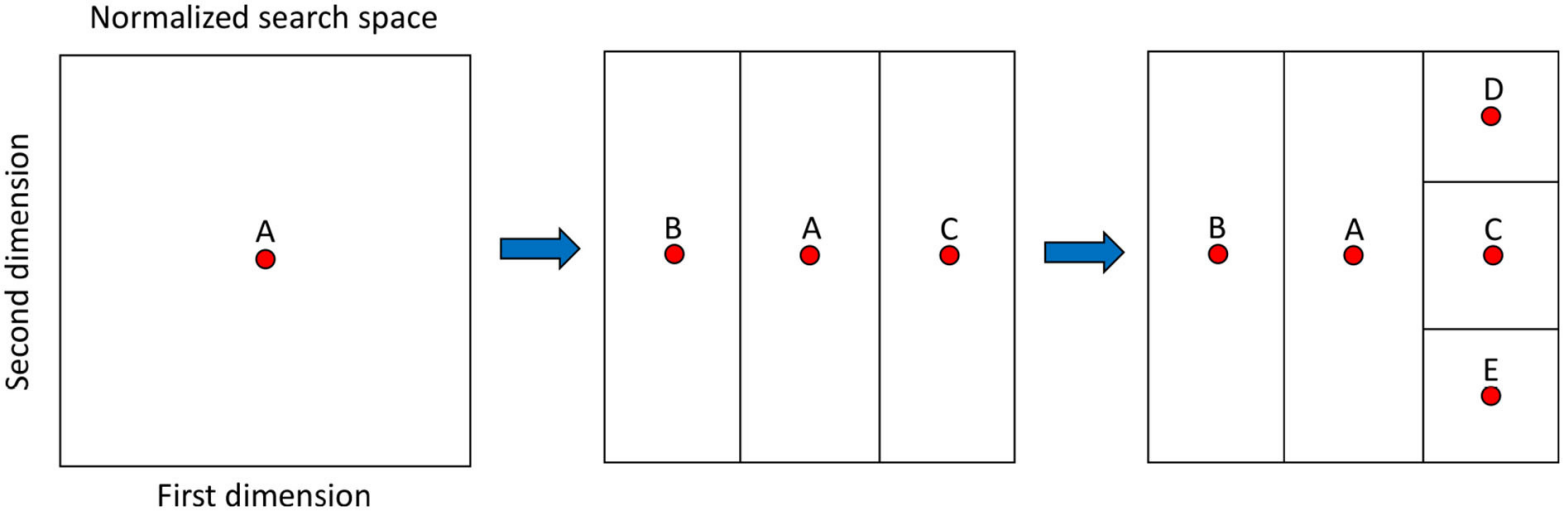
Main aspects of dividing rectangles (DIRECT) and its search space after two iterations. At the beginning of the initial iteration, point A is the first evaluated and represents the first and only rectangle (square) defined by the search space. It is trisected and results in the rectangles defined by points B and C. At the second iteration, the rectangle defined by point C is selected and trisected resulting in rectangles defined by points D and E.

More specifically, DIRECT starts by normalizing each variable to [0, 1] so that the search space becomes the unit hypercube. Then, the method proceeds by dividing it into sub-rectangles. This scheme determines the name of the method, since DIRECT comes from ‘DIviding RECTangles'. The rectangles are represented by the value of the objective function at their center, which avoids the effect of problem dimensionality: rectangles only have one center independently of the dimensions. It is also relevant to highlight that the referred division is a trisection in reality, which allows keeping the focus on the original rectangle without further re-evaluation, i.e., its center stills refer to a different region. Figure 4 depicts these ideas assuming a 2D search space and two divisions (trisections).

The fundamental aspect of DIRECT is how the rectangles are selected for division and further exploration at each iteration. This selection is deterministic and theoretically considers every possible balance between exploration and exploitation (Lipschitz-like constant). As detailed in Jones (2001), a pure global method would always select the widest rectangle. A pure local one would opt for the one with the best value at its center. The former avoids overlooking the promising regions, while the latter promotes that.

**Figure 5:**
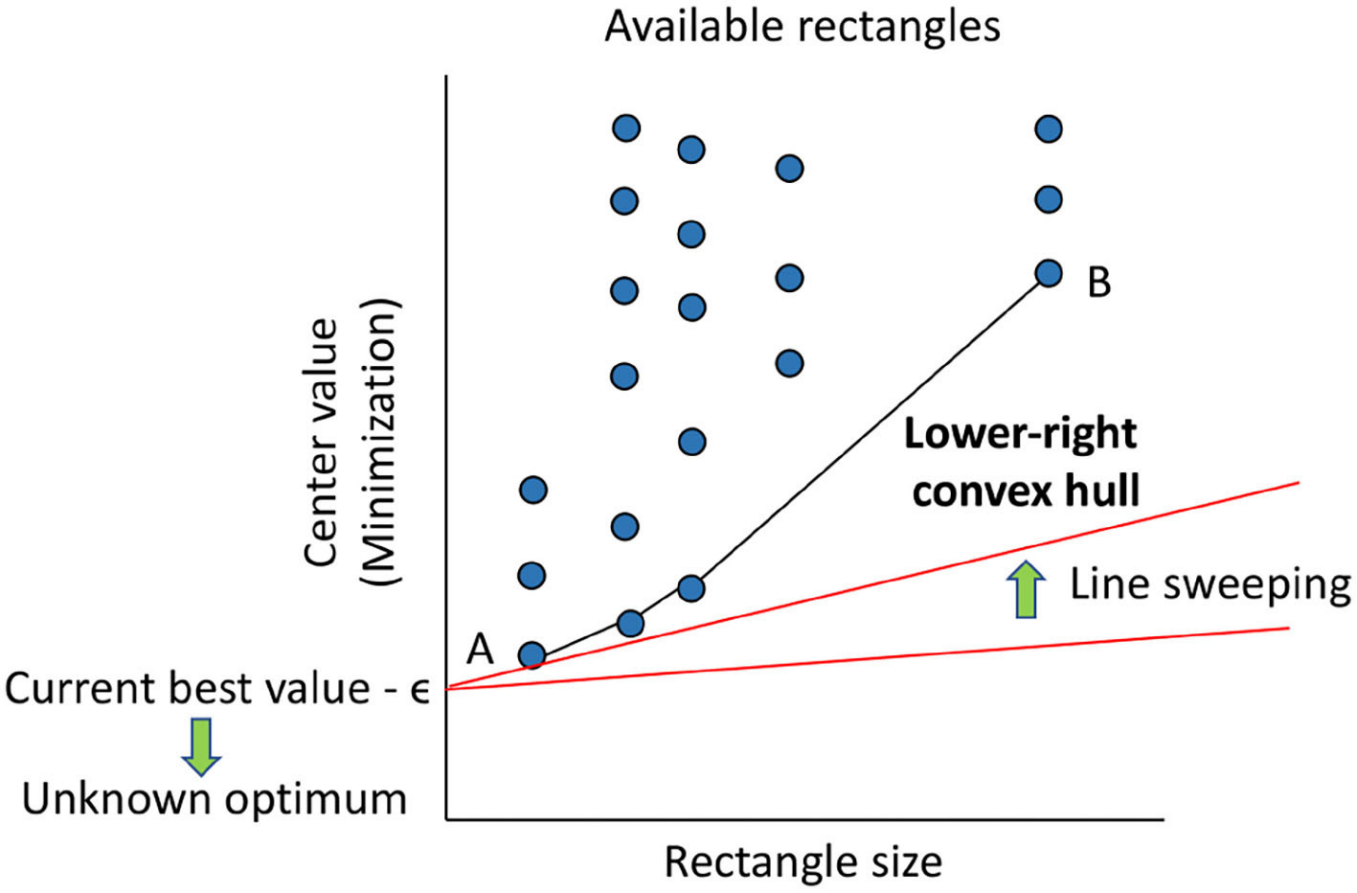
Selection of rectangles to further explore (divide) in DIRECT for a hypothetical minimization problem. The available rectangles are represented as dots (circles are shown for clarity). The horizontal axis corresponds to the size of each rectangle, and the vertical one shows the value of the objective function at its center (lower is better for minimization). In this context, rectangle A is the one with the best central value, while B encompasses the broadest region. The rectangles selected will lie in the lower-right convex hull, which represents the optimal balance between exploration and exploitation. The red lines at the bottom show an alternative way to derive this selection method: a line is anchored at every value better than known, starting from that one minus the desired accuracy (minimum relevant change), ϵ, and swept upwards until reaching a rectangle. Repeating the process to (negative for minimization) infinity results in the same convex hull (and avoids regions with expected negligible improvements).

DIRECT does not force itself to select just one rectangle, which would require parameters to tune. Instead, the method computes all the weightings of local vs. global search. For this purpose, it defines the size of any rectangle as the distance between its center and one of its vertices. Then, for every selection at a particular iteration, the method represents all the available rectangles depending on their size and the value of their center. After that, it proceeds to select those in the low-right convex hull. Figure 5 depicts this idea assuming a minimization problem. The selected rectangles are the balanced options between local and global search considering the central value and size of the corresponding regions. Notice the similitude of this approach to computing the Pareto set as the solution to a multi-objective optimization problem (Filatovas et al., 2017).

Interestingly, as explained in Jones (2001) and also shown in Figure 5, the selection of rectangles can be alternatively derived from the rate of change of the function in each. If one knows the optimal value, anchors a half-line at it, and swings the free extreme upwards, the first dot touched represents the rectangle with the most reasonable rate of change, i.e., gradual instead of steep, to contain the optimum. Hence, that rectangle must be selected. In reality, the optimal value is not usually known. However, it is possible to repeat this process from the best value known so far, as the optimal value will be equal to or lower than it, to minus infinity. Selecting the touched dot for each anchored point results in the lower-right convex hull previously defined. It is hence possible to obtain the same selection scheme yet by thinking differently. Besides, it is possible to subtract an arbitrary value, ϵ, to the best value known to discard from the hull the rectangles with negligible improvements. Accordingly, DIRECT only expects as input the maximum number of function evaluations and the constant ϵ, which can be seen as the desired accuracy of the solution. The interested reader can refer to Jones (2001) for further information about this algorithm.

Despite its good properties (conceptual simplicity, ingenious deterministic exploration, and requiring a single parameter), DIRECT is not free of potential drawbacks, and researchers have proposed numerous variants (Jones and Martins, 2021; Stripinis and Paulavičius, 2022). The two main flaws of the initial method are (Stripinis and Paulavičius, 2022) i) the potential waste of function evaluations in sub-optimal regions for functions with many local optima and ii) the slow convergence rate even after having identified the basin of the global optimum (Jones and Martins, 2021). Accordingly, the method selected for this work is one of the revised versions of DIRECT, i.e., DIRECT-GL, which tries to overcome both (Stripinis et al., 2018).

For this purpose, the authors of DIRECT-GL modified the selection of rectangles to consider more than its ancestor. The process has two stages and fits into the original framework without requiring extra parameters. The first one enhances the global search component of the method, represented by the letter ‘G' in its name. It starts by adding the rectangles with the best central value and prioritizing those that are bigger. This approach results in more rectangles of medium size and the best values. The second phase is similar, but it considers the Euclidean distance to the best point known so far instead of the objective function value. Namely, it tries to add more hyper-rectangles close to the current minimum. This strategy strengthens the exploration of the most promising area, i.e., it enhances the local search aspect of the method, represented by the letter ‘L' in its name.

Aside from the computational studies in Stripinis et al. (2018), the effectiveness of this strategy is supported by the recent comparison in Stripinis and Paulavičius (2022), where DIRECT-GL exhibits the best performance among all the DIRECT-based methods. The implementation in Stripinis and Paulavičius (2022), also used in this work, unifies the results of both stages in a single selection. This aspect differs from the original work to make the method more suitable for parallelization and more effective.

#### 2.3.4. Random search

A pure random search procedure is arguably the simplest global optimizer (Brooks, 1958), and it belongs to the stochastic family of optimization methods (Cruz et al., 2018). More specifically, it consists of randomly generating solutions in the search space while keeping a record of the best one found so far. Algorithm 2 describes this process in detail. Notice that it is expressed in general terms, and the comparison criterion is dependent on whether the objective function is to be minimized or maximized.

**Algorithm 2:** Random search

**Require:** Objective function: *f*, Evaluations allowed: *evals*
1: solution ← ∅
2: currentVal ← worst value
3: iter ← 0
4: **while** iter < evals **do**
5: point ← random()
6: **if** *f*(point) is better than currentVal **then**
7: solution ← point
8: currentVal ← *f*(point)
9: **end** **if**
10: iter ← iter + 1
11: **end** **while**
12: **return** solution

Despite its simplicity, this method converges to a global optimum when the number of allowed evaluations tends to infinity (Brooks, 1958). On the one hand, its practical applicability is low due to the lack of orientation during the search. For this reason, it has been initially selected for the problem at hand as the expected baseline reference, especially considering that the computational cost of the objective function makes it difficult to work with high evaluation budgets. Thus, the previous methods are expected to outperform this one because of their sophisticated components to explore and exploit the search space (Van Geit et al., 2008). On the other hand, the simple structure of this procedure, which is also embarrassingly parallel in terms of high-performance computing (Trobec et al., 2018), ensures a high rate of solution evaluations in an appropriate computing platform. Hence, its results can be of interest depending on the ultimate problem difficulty and the quality requirements.

## 3. Experimentation and results

### 3.1. Problem-specific setup and reference value

A sample tuning problem of a spiking neural model of striatum plasticity has been addressed to assess the performance of the considered optimization methods. The problem was selected due to its high number of parameters, biological relevance, and computational cost of evaluating solutions. The model details are in Gonzalez-Redondo et al. (2022), and the important problem-specific setup is summarized below.

The simulation time was 500 s, enough for the hand-tuned models to converge to a solution. The model contains 2,000 LIF input neurons and 16 spiking LIF output neurons with an adaptive threshold divided into two channels (one per possible action). During the learning protocol, five different repeating stimuli were used, besides noise. The duration of each stimulus is taken from a uniform random distribution between 100 and 500 ms. Five different random seeds were used for every set of parameters tested and the resulting fitness of each seed averaged.

The best result obtained without optimization methods is shown in Figure 1. Panels C-F shows network activity and rewards/punishments during the last 5 s of simulation. The most relevant information is accuracy through the training process. The mean of the last 100 s of the accuracy is used as the fitness for the objective function. The procedure to calculate the accuracy is described in Section 2.1. The accuracy evolution of the best result obtained by an expert after manually tuning for 2 months the parameters using a trial-and-error procedure is shown in Figure 1G. Good sets of parameters typically plateau after 400 s, as wrong actions are taken from time to time even with further training.

### 3.2. Computational setup

The computational platform used belongs to the high-performance computing cluster of the Supercomputing—Algorithms research group from the University of Almería, Spain. Specifically, up to 8 Bull Sequana X440-A5 nodes were used to launch different executions. Every node features 2 AMD EPYC Rome 7,642 with 48 cores each, i.e., 96 cores in total, 512 GB of RAM, and 240 GB SSD as its main disk. In a core of one of these nodes, evaluating a candidate solution or set of model parameters for the configuration described above takes 1.78 ± 0.12 h on average. This value has been computed by generating and evaluating 96 random feasible parameter sets. The software environment consists of Matlab 2020b for SurrogateOpt, DIRECT-GL, Random Search, and Python 3.6.8 for RBFOpt.

Two computational budgets have been considered, 300 and 600 function evaluations. The first results from taking into account the estimated run time as follows: Since each evaluation takes 1.78 h on average using a high-end processor, 300 evaluations should take 300 × 1.78 = 534 h at least, i.e., 22 days approximately. This estimation assumes a sequential execution workflow, as a human expert will likely proceed, and neglects the overhead associated with the internal computations of the optimizers. The value of 22 days of work is in the same order of magnitude as the most favorable conditions found by the authors of this paper when doing the referred model fitting by hand. Similarly, the value of 300 function evaluations is equal to the budget used by RBFOpt by default. Concerning the second limit used, 600, it has been adjusted to two times the lower value. By proceeding this way, it will be possible to assess the benefits of doubling the effort. This value would also be close to the maximum function evaluations that Surrogateopt would assign to the problem at hand, namely, 50 × 13 = 650 according to the official documentation.

The sequential run time estimations would be 22 and 44 days, approximately. The former, for 300 evaluations, was demanding, but the latter, for 600, started to be overwhelming for a person. Nevertheless, when automating the process by using optimizers compatible with parallel computing and there is access to a cluster, both conditions met in this work, the run time can be significantly lower. Ideally, by deploying 96 threads, the objective function evaluation time could be reduced by a factor of up to 96. This speedup would mean turning the 534 h turn into 5.56, approximately. In general, the perfect speedup is achieved rarely. Spawning and managing concurrent execution units comes at a cost, sequential tasks do not benefit from them, and there might not always be enough work for all (e.g., few hyper-rectangles selected by DIRECT-GL at a particular iteration). Regardless, the time taken by the optimizers in the cluster is expected to be significantly lower than estimated above for sequential execution.

Apart from controlling the number of function evaluations allowed, the four solvers were configured with their default options. This included configuring RBFOpt to use the Bonmin (Bonami et al., 2008) and Ipopt (Wächter and Biegler, 2006) solvers (Costa and Nannicini, 2018) for addressing the internal sub-problems that arise (e.g., adjusting the radial basis function interpolants). Aside from this, notice that RBFOpt stands out by being capable of using a less accurate yet faster version of the objective function. Working with it requires both the referred kind of function and the lower and upper bounds of the expected error. To accelerate the neural model assessment, i.e., the objective function, the number of simulation seeds has been reduced from 5 to 1, which should make its computation five times faster on average.

The inaccuracy estimation has been computed as follows: 8 cluster nodes with the same specifications as defined above were used to generate 26,880 feasible configurations randomly. Then, the SD of the objective value for each one of the five simulation seeds was recorded. The average SD between seeds for the same configuration was approximately 0.03. Then, according to the empirical rule of Statistics, this average SD was multiplied by 3 to cover 99.7% of the values assuming a standard distribution. The result is 0.09, which was ultimately rounded up to 0.10 to add an arbitrary extra margin. Accordingly, the inaccurate yet faster function is passed to RBFOpt considering that the real value (if 5 simulations seeds were considered instead of 1) will be in the range of ±0.10 plus the inaccurate estimation.

### 3.3. Numerical results

Table 3 contains the results for the model tuning problem addressed with each optimizer and function evaluation budget. The first column shows the optimization algorithm. The second one displays the number of function evaluations allowed. The values generally refer to the standard function with five simulation seeds. However, the two last cases of RBFOpt combine the full function with the one featuring a single simulation seed to be faster despite reducing its accuracy. They include the word ‘fast' to highlight this aspect. It is noted that dividing the fast term by 5 and adding it to the standard one results in the same budgets considered, i.e., 300 and 600 standard function evaluations. In the beginning, the second configuration of this type for RBFOpt consisted of 400 complete evaluations and up to 1,000 fast ones, but the results were worst, and the solver opted for not executing that many fast evaluations. Thus, it seems preferable to put more emphasis on complete ones even though the estimated cost is theoretically equivalent, and we ultimately chose the configuration shown.

**Table 3:** Performance metrics for each optimizer and configuration considered computed with the results of 20 independent executions.

**Optimizer**	**Function** **evaluations**	**Average** **fitness**	**Standard** **Deviation**	**Confidence** **Interval (95%)**	**Average run** **time (h)**
SurrogateOpt	300	0.7269	0.0667	[0.6957, 0.7581]	6.07
	600	**0.7699**	0.0691	[0.7376, 0.8022]	11.26
RBFOpt	300	0.5325	0.1461	[0.4641, 0.6009]	6.85
	600	0.6267	0.1434	[0.5596, 0.6938]	13.27
	200 + 500 fast	0.5843	0.1458	[0.5161, 0.6525]	5.30
	500 + 500 fast	0.5923	0.1550	[0.5198, 0.6648]	12.29
DIRECT-GL	300	0.4165	-	[0.4165]	81.98
	600	0.4618	-	[0.4618]	103.38
Random Search	300	0.5492	0.1412	[0.4831, 0.6153]	5.56
	600	0.6159	0.1027	[0.5678, 0.6640]	11.13

The following two columns contain the average efficiency (higher is better, with the best value in bold font) and the SD for each optimizer and configuration. All the stochastic methods have been independently executed 20 times. With this information, the 95% confidence intervals have been computed according to the *t*-Student distribution considering the sample sizes (i.e., under 30 records each). They are shown in the fifth column. The sixth and last columns contain the average run time for each case (the SD are omitted because of not being either significant or especially relevant for this variable). For DIRECT-GL, the run times have been obtained by launching 8 independent executions, one per available node. Finally, it is relevant to mention that the RandomSearch results have been obtained from the dataset with 26,880 random points used to assess the accuracy of the fast version of the objective function. More specifically, they come from taking 20 random samples with as many instances as the function evaluation budget. Thus, the run times of this method have been analytically estimated by multiplying the average evaluation time by the computational budget. They were ultimately divided by the number of CPU cores due to the embarrassingly parallel nature of the process.

Concerning the results, the most noticeable aspect was that DIRECT-GL showed the worst performance in terms of achieved fitness and required run time. The aptitude of its solutions for 300 and 600 function evaluations is even worse than that obtained with the simplest method, i.e., RandomSearch. Both results, i.e., 0.4165 and 0.4618, stay outside of the confidence interval of this stochastic method, and below the lower bounds for 300 and 600 function evaluations. The same occurs when considering RBFOpt and its configuration with 300 evaluations. Accordingly, the difference between these methods is statistically significant. Its average run time is also significantly higher than the rest, which comes from the fact that the parallelism of DIRECT-GL is strictly bounded by the number of selected rectangles at any point. For this reason, it will not always exploit all the available CPU cores, which is critical in the context of interest.

Conversely, SurrogateOpt stands out as the best-performing method in terms of achieved fitness and a low SD. The lower bound of its lowest confidence interval does not fall into the range of any other one, so the observed difference between these optimization strategies for the target problem is significant. Besides, considering that the run time of RandomSearch is an optimistic approximation, it could be said that the computational performance is virtually equivalent. Accordingly, the direct conclusion that can be drawn from the results shown in Table 3 is that SurrogateOpt is the best solver for this kind of model-tuning problem. Moreover, its average results are comparable to that obtained by an expert after a tedious and time-demanding model tuning process. More specifically, as detailed in Section 3.1, the fitness of the expert-based model-tuning was 0.7216, while the average of SurrogateOpt is 0.7269 with 300 function evaluations only. Nevertheless, one can doubt the effectiveness of doubling the computational budget for SurrogateOpt because the confidence intervals of both cases overlap.

When confidence intervals do not overlap, the difference between the two groups is statistically significant. However, when they do, the difference might still be relevant Goldstein and Healy (1995), Sullivan (2008). To avoid this uncertainty, the confidence intervals for their difference will be computed. For this purpose, as both samples have less than 30 instances, the *t*-Student distribution will be used again. Notice that the following formulation assumes similar variances in the population, as it occurs between both cases of SurrogateOpt. The pooled estimate of the common SD, *S*_*P*_ is computed as follows:


(8) $$\begin{array}{l} S_{P}=\sqrt{\frac{\left(n_{1}-1\right)\sigma_{1}^{2}+\left(n_{2}-1\right)\sigma_{2}^{2}}{n_{1}+n_{2}-2}} \end{array}$$

where *n*_1_ and *n*_2_ are the sample sizes of groups 1 and 2, respectively, and σ_1_ and σ_2_ refer to their corresponding SD. With this information, the confidence interval for the difference between two means, ${\bar{x}}_{1}$ and ${\bar{x}}_{2}$, is obtained as follows:


(9) $$\begin{array}{l} \left({\bar{x}}_{1}-{\bar{x}}_{2}\right)\pm tS_{P}\sqrt{\frac{1}{n_{1}}+\frac{1}{n_{2}}} \end{array}$$

where *t* refers to the appropriate value from the *t*-Student table (determined by the sample sizes, as introduced) for the desired confidence level and *n*_1_ + *n*_2_ − 2 degrees of freedom. It is noticed that the two terms after *t* define the standard error of the difference in means between ${\bar{x}}_{1}$ and ${\bar{x}}_{2}$.

Back to the mean and SD of both configurations of SurrogateOpt, the 95% confidence interval of their difference is [-0.0865, 0.0005] according to 9. It defines a range of likely values for the difference in means between both cases, ${\bar{x}}_{1}$ - ${\bar{x}}_{2}$, where ${\bar{x}}_{1}$ is that of 300 function evaluations and ${\bar{x}}_{2}$ is that of 600. Theoretically, since the interval contains the null value, i.e., 0, it can be concluded that there is no statistically significant difference between the average results of launching SurrogateOpt with 300 and 600 function evaluations. Even if the upper bound of this interval were slightly below zero, the average difference could be perceived as negligible yet confirmed. That said, in practical terms, since the outcome of this process is a model parameter set with the highest possible fitness, it seems reasonable to work with 600 function evaluations and several independent runs whenever possible.

Regarding RBFOpt and RandomSearch, they remain between the best performing solver, SurrogateOpt, and the worst, DIRECT-GL. Being free to use without cost, unlike SurrogateOpt, is their main attribute in this context. It is hard to find the best option among them at a glance due to the significant overlap and comparatively high SD. Technically, RBFOpt offers the best average results when allowed to execute 600 function evaluations. Besides, the 95% confidence interval of the difference between 300 and 600 function evaluations confirm the effectiveness of doubling the computational effort, yet it is arguably negligible. However, the difference with the cases using fast evaluations is not statistically significant. The same occurs when comparing RandomSearch and RBFOpt with 600 function evaluations. This close similarity indirectly benefits RandomSearch, as it is the simplest strategy to apply, especially when there is an available parallel computing platform.

### 3.4. Insight into optimization-based model tuning results

**Figure 6:**
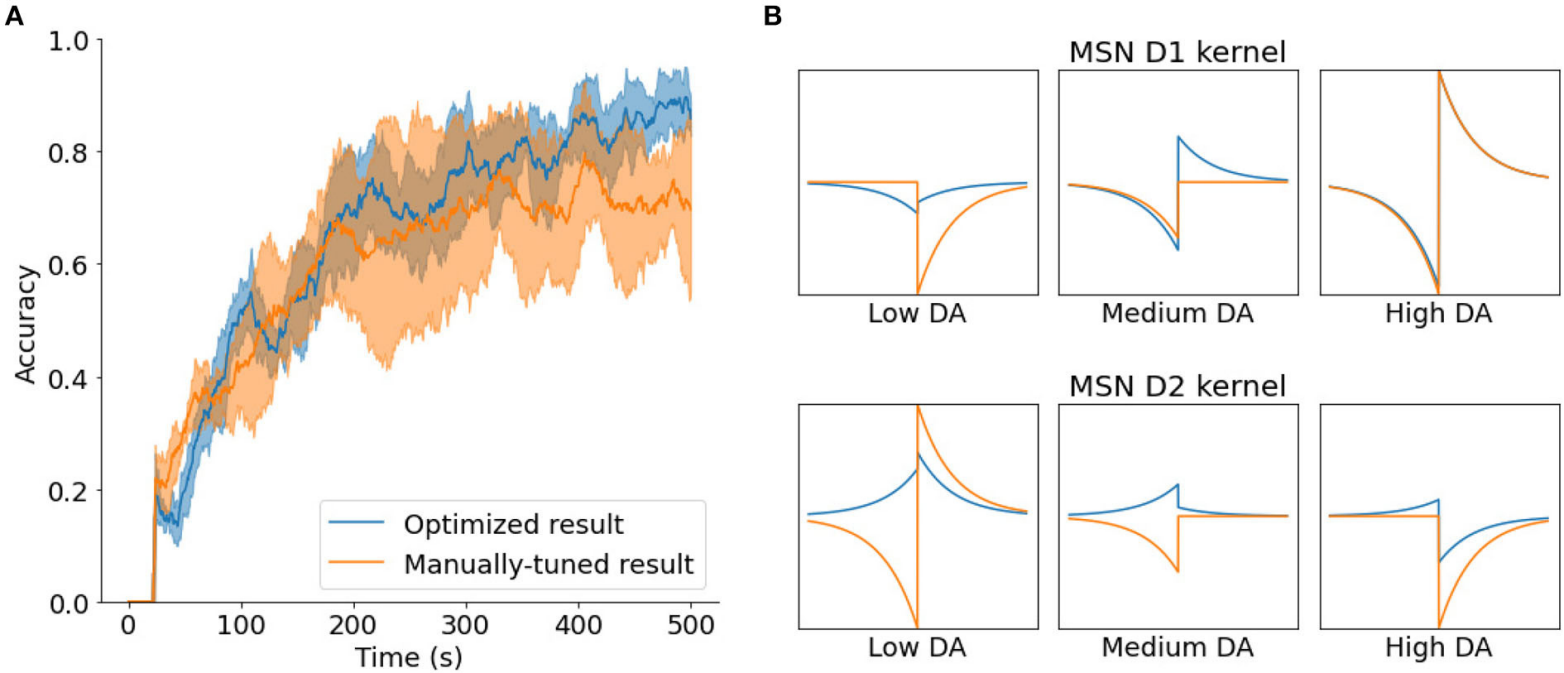
**(A)** Comparison between one of the best results obtained with the SurrogateOpt optimization method (blue) with the best manually-tuned result (orange), with the mean and SD (*n* = 5). **(B)** Comparison of parameters related with the STDE kernel.

The results of the optimization process yielded better learning capabilities than those of manual tuning. Figure 6A shows one of the best configurations found by SurrogateOpt, the preferred method, in action, and compares it to the manually tunned option. Although the manually tuned result plateaus after 400 s, the optimized result continues to improve accuracy after that point. In addition, the optimized result is more reliable, as its SD is smaller.

Of the various parameters, the most interesting are the ones that define the shape of the STDE kernels of the learning rule for the neurons (MSN D1 and D2). Figure 6B shows the differences between the manually tuned kernel and the optimized kernel. While the manually tuned solution tends to use asymmetrical kernels in every case, it seems that the optimized solution uses symmetrical kernels for low DA and asymmetrical kernels for high DA.

If we considered symmetrical kernels having values with equal signs and asymmetrical kernels with opposite signs, this is in accordance with the values obtained by Gurney et al. (2015) in their exhaustive parametric search (Figure 11 in their article). This could be relevant as they are considering more biological constraints than this study. The only discrepancy is in the case of high DA in MSN D2 neurons, where the optimized kernel is reversed from the range obtained by Gurney et al. However, further research is needed to better understand the significance of these findings and the plausibility of the proposed parameters.

## 4. Conclusions and future work

This study addresses the tuning of spiking neural models of striatum plasticity by using state-of-the-art black box and surrogate optimization methods. This kind of model is useful for understanding how the brain could perform online RL, a fundamental ability that is essential for many tasks such as motor control or decision-making. However, tuning these models is a difficult task due to the high dimensionality of the parameter space and the time required for the simulations. In addition, experts are often biased in their choices of parameter values. This problem can be addressed as an optimization problem, which can be solved using different methods.

This study makes a selection of optimization algorithms designed for computationally-demanding objective functions and compatible with parallel computing. The goal is to find the best alternative to avoid the necessity of tedious and expert-biased trial-and-error tuning of biologically realistic neural models that require much time to be simulated. This approach could automate model tuning despite not having been broadly studied yet in this context. Automation will not only avoid potential errors and biased configurations, but it can also reduce the required time from weeks to hours.

The solvers considered are SurrogateOpt, shipped with the Optimization Toolbox of Matlab, RBFOpt, which is an open-source optimizer written in Python, and DIRECT-GL, which is an improvement of a widespread optimizer written in Matlab yet open-source too. Aside from them, a naive pure-random search strategy has also been implemented. They have been compared when trying to tune a spiking neural model of striatum plasticity that takes 1.78 h on average to be simulated in the computing platform. The methods were only allowed to evaluate 300 solutions in the first case and 600 in the second. Both computational budgets were in the same order of magnitude as a human expert takes to tune the model used as the benchmark, i.e., several hundred function evaluations.

SurrogateOpt stands out as the best solver to use, and it is hence recommended for this kind of computationally demanding neural model tuning problem. It achieves the best average results with the lowest SD and significantly distinguishes itself from the rest. The model configurations that it finds with 300 function evaluations can compete with the expert-based reference. Namely, the fitness of the expert-based model tuning was 0.7216 after 2 months of work, and the average of SurrogateOpt is 0.7269 with 300 function evaluations (6 h approximately). This average increases up to 0.7699 when the method can launch 600 evaluations. However, the effectiveness of doubling the computational effort could not be confirmed on average for the studied problem. Regardless, the generic recommendation made is to work with the highest computational budget and multiple independent executions due to its stochastic nature.

RBFOpt and RandomSearch, both stochastic methods too, perform significantly worse than SurrogateOpt in terms of average fitness despite spending similar times. Hence, they should be only used when there is no access to the referred solver. Nevertheless, the potential of RandomSearch for this kind of problem is remarkable, especially when a high-performance computing platform is available. This method is trivial to implement, and its performance can be significantly improved by increasing the number of evaluated solutions per unit of time.

In contrast to the rest, DIRECT-GL, the only deterministic solver chosen, is also the worst option for the problem at hand. Its parallel computing capabilities are limited by the number of promising regions that the method can find. Since it does not find the best regions in the search space and finds few attractive zones, the algorithm is unable to fully exploit the computing platform. These aspects make it not only the solver that achieves the worst tuning configurations but also the slowest one.

In future work, the best-performing solver will be used to tune other neural models featuring computationally demanding simulation processes. Additionally, the study might be extended as new suitable methods arise.

## Data availability statement

The original contributions presented in the study are included in the article/Supplementary material, further inquiries can be directed to the corresponding author.

## Author contributions

NC and AG-R conceived and designed the study and wrote the first version of the manuscript. AG-R developed the neural model and manually tuned it, while NC linked the model to the optimization methods selected. JG, AG-R, and EO did the literature review concerning neural model tuning and studied the results in terms of neuroinformatics and checked the quality of the optimization-based configurations. NC, JR, and PO did the literature review regarding optimization algorithms and analyzed and discussed the performance of each optimizer. JG, JR, EO, and PO revised it and suggested improvements. All the authors made relevant contributions to the article and approved the submitted version.

## Funding

This research has been funded by the R+D+i projects RTI2018-095993-B-I00 and PID2021-123278OB-I00, financed by MCIN/AEI/10.13039/501100011033/ and ERDF A way to make Europe; by the Junta de Andalucía with reference P18-RT-1193 and by the University of Almería with reference UAL18-TIC-A020-B. NC is supported by the Ministry of Economic Transformation, Industry, Knowledge and Universities from the Andalusian government. This research was supported by the Spanish Grant INTSENSO (MICINN-FEDER-PID2019-109991GB-I00), Regional grants Junta Andalucía-FEDER (CEREBIO P18-FR-2378). This research had also received funding from the EU Horizon 2020 Framework Program under the Specific Grant Agreement No. 945539 (Human Brain Project SGA3). Finally, AG-R was supported by an FPU Fellowship from the Spanish Ministry of Education (FPU17/04432).

## Conflict of interest

The authors declare that the research was conducted in the absence of any commercial or financial relationships that could be construed as a potential conflict of interest.

## Publisher's note

All claims expressed in this article are solely those of the authors and do not necessarily represent those of their affiliated organizations, or those of the publisher, the editors and the reviewers. Any product that may be evaluated in this article, or claim that may be made by its manufacturer, is not guaranteed or endorsed by the publisher.
