# Supplementary material for: Black-box and surrogate optimization for tuning spiking neural models of striatum plasticity
Source: Front Neuroinform. 2022 Oct 20;16:1017222. doi: 10.3389/fninf.2022.1017222 (PMC9630480; doi:10.3389/fninf.2022.1017222)
Supplement: Supplementary file 2 [file Data_Sheet_2.zip › Appendix-Black_box.pdf]

Appendix: Black-box and surrogate optimization for tuning spiking neural models of striatum plasticity

N.C. Cruz [1], **A. González-Redondo** [1,*], **J.L. Redondo** [2], **J.A. Garrido** [1], **E.M. Ortigosa** [1] **and P.M. Ortigosa** [2]

[1] *Dept. of Computer Engineering, Automation and Robotics, University of Granada, Granada, Spain*
[2] *Dept. of Informatics, University of Almería, ceiA3 Excellence Agri-food Campus, Almería, Spain*

Correspondence*:
A. González-Redondo
alvarogr@ugr.es

SUPPLEMENTARY MATERIALS

Neuron models and parameters used

We used conductance-based versions of the Leaky-Integrate and Fire (LIF) neuron model (Gerstner and Kistler, 2002) in every layer of the network, but with different parameters. We classify the neuron types according to the layer they belong to: cortical neurons for the input, striatal neurons for the learning layer, and action neurons for the output. There is also a dopaminergic neuron that receives the rewards and punishments.

The parameters used for each type were manually tuned to obtain reasonable firing rates. For the cortical neurons we used a number of spikes per input cycle (with 8 cycles per second) close to Masquelier et al. (2009) and Garrido et al. (2016). For the striatal neurons, we tuned the parameters to obtain a mean firing rate of around one spike per second to be within biological ranges (Miller et al., 2008) but with activity peaks of two or three spikes per input cycle (16-24 spikes per second). The action neurons are tuned to fire every input cycle if they receive enough stimulation from its channel (at least two more spikes from D1 neurons than D2 neurons each cycle). The dopamine neuron was tuned to have a firing range from 50 to 350 spikes per second. The parameters used for each neuron type are shown at supplementary Table 1.

Parameter	Cortical	Striatal	Action	Dopaminergic
e_{exc} (mV)	0.0	0.0	0.0	0.0
e_{inh} (mV)	-85.0	-85.0	-85.0	-85.0
τ_{AMPA} (ms)	5.0	5.0	5.0	5.0
τ_{GABA} (ms)	10.0	30.0	60.0	10.0
τ_{ref} (ms)	1.0	15.0	15.0	1.0
C_m (pF)	250.0	50.0	100.0	250.0
g_{leak} (nS)	25.0	10.0	25.0	25.0
V_{thr} (mV)	-40.0	-50.0	-40.0	-65.0
e_{leak} (mV)	-65.0	-65.0	-65.0	-40.0

Table 1. Neuron parameters used in the model.

REFERENCES

Garrido, J. A., Luque, N. R., Tolu, S., and D'Angelo, E. (2016). Oscillation-Driven Spike-Timing Dependent Plasticity Allows Multiple Overlapping Pattern Recognition in Inhibitory Interneuron Networks. *International Journal of Neural Systems* 26, 1650020. doi:10.1142/S0129065716500209

Gerstner, W. and Kistler, W. M. (2002). *Spiking neuron models: Single neurons, populations, plasticity* (Cambridge University Press)

Masquelier, T., Hugues, E., Deco, G., and Thorpe, S. J. (2009). Oscillations, Phase-of-Firing Coding, and Spike Timing-Dependent Plasticity: An Efficient Learning Scheme. *Journal of Neuroscience* 29, 13484–13493. doi:10.1523/JNEUROSCI.2207-09.2009

Miller, B. R., Walker, A. G., Shah, A. S., Barton, S. J., and Rebec, G. V. (2008). Dysregulated information processing by medium spiny neurons in striatum of freely behaving mouse models of huntington's disease. *Journal of neurophysiology* 100, 2205–2216
