# Supplementary figures and images for: Black-box and surrogate optimization for tuning spiking neural models of striatum plasticity
Source: Front Neuroinform. 2022 Oct 20;16:1017222. doi: 10.3389/fninf.2022.1017222 (PMC9630480; doi:10.3389/fninf.2022.1017222)

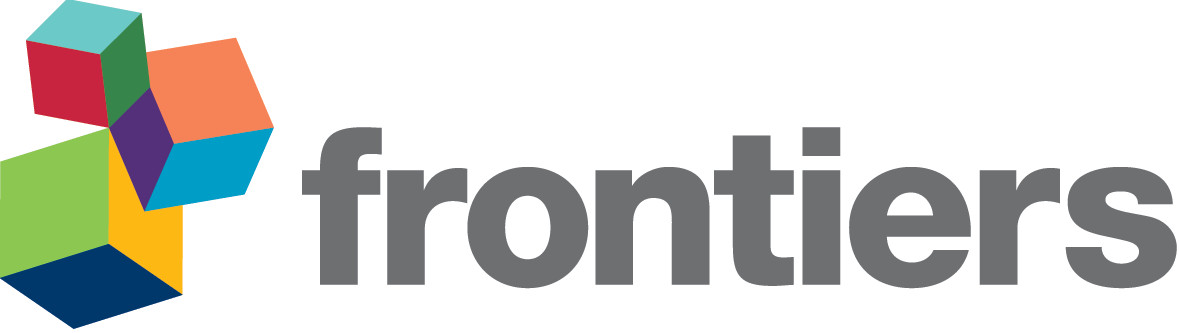

Supplement: Supplementary file 2 [file Data_Sheet_2.zip › logo1.jpg]
